# Conditions for successful implementation of couple-based collaborative management model of diabetes among community-dwelling older Chinese: a qualitative comparative analysis

**DOI:** 10.1186/s12877-023-04565-y

**Published:** 2023-12-11

**Authors:** Jing Zhang, Conghui Yang, Yixuan Liu, Dadong Wu, Lingrui Liu, Huiqiong Zheng, Dong (Roman) Xu, Jing Liao

**Affiliations:** 1 Department of Medical Statistics & Epidemiology, Sun Yat-sen Global Health Institute, School of Public Health, Institute of State Governance, Sun Yat-sen University, No.135 Xingang West Road, Guangzhou, 510275 P.R. China; 2 Sun Yat-sen Global Health Institute, School of Public Health, Institute of State Governance, Sun Yat-sen University, Guangzhou, P.R. China; 3 Affiliated Shenzhen Maternity & Child Healthcare Hospital, Southern Medical University, Shenzhen, P.R. China; 4 Center for Methods in Implementation and Prevention Science, Yale School of Public Health, New Haven, United States; 5 Center for World Health Organization Studies, Department of Health Management, School of Health Management, Southern Medical University, Shenzhen, P.R. China; 6 ACACIA Lab for Implementation Research, SMU Institute for Global Health (SIGHT), Dermatology Hospital of Southern Medical University (SMU), Guangzhou, P.R. China

**Keywords:** Qualitative comparative analysis, Older people, Couple-based management, Community management

## Abstract

**Background:**

Diabetes mellitus is a prevalent and potentially devastating chronic illness affecting many older adults. Given spousal involvement in many aspects of diabetes management, coping with their partners is increasingly seen as a potential solution to make up for limited resources. This study aimed to identify the key conditions for optimal implementation of couple-based collaborative management model (CCMM) among Chinese older couples with type 2 diabetes mellitus.

**Methods:**

Older couples and community healthcare practitioners were selected according to couples’ joint intervention attendance rate and community’s average attendance rate. This mixed methods research consisted of a qualitative phase and a quantitative phase. In the qualitative phase, in-depth interviews were conducted among 12 pairs of couples in the intervention group and 4 corresponding practitioners, in the follow-up period of the multicentered RCT from January to April 2022. Qualitative comparative analysis (QCA) in the quantitative phase to identify conditions influencing CCMM’s implementation and to explore necessary and sufficient combinations of conditions (i.e., solutions) for improving patients’ glycated hemoglobin (HbA1c) control (outcome).

**Results:**

Key conditions included implementation process, couple’s role in diabetes management, their belief and perception of CCMM, as well as objective obstacles and subjective initiative for behavior change. Accordingly, major barriers in CCMM’s implementation were patients’ strong autonomy (particularly among husbands), misbelief and misperception about diabetes management as a result of low literacy, and mistrust of the practitioners. QCA further revealed that no single condition was necessary for effective HbA1c control, while three types of their combinations would be sufficient. Solution 1 and 2 both comprised the presence of spousal willingness to help, plus correct belief and perception of diabetes management, well embodying the utility of couple collaborative management in supporting patients’ HbA1c control. On the other hand, solution 3 indicated that high-quality implementation even without spousal support, can promote the patient’s subjective initiative to overcome objective obstacles, suggesting enhanced self-management for HbA1c control.

**Conclusions:**

Tailored CCMM should be implemented in reference to older couple’s preferences and literacy levels, to ensure intervention fidelity, and establish correct understanding of collaborative management among them.

**Supplementary Information:**

The online version contains supplementary material available at 10.1186/s12877-023-04565-y.

## Introduction

Community-based disease management is an important healthcare delivery strategy for chronic diseases such as diabetes mellitus [1]. It involves community engagement in defining the problem and develops partnerships to implement management strategies [2]. In the past three years, considerable community healthcare resources have been devoted to the prevention and control of COVID-19 in China [3], resulting in overlooking many other health issues including chronic disease management. Increased attention thus has been paid to family involvement as a protentional solution to compensate the limited resources [4]. Increasing evidence has suggested that family members significantly improved the self-management activities of older people [5]. Cooperative management of family members, particularly spouses, have great influences on patients’ blood sugar control, including physical activities, dietary behaviors, medication adherence, and glucose monitoring [6–8].

The couple-based collaborative management model (CCMM) was proposed as a solution to better engage family in older patients’ care [9]. CCMM utilizes the interdependence of patients and their spouses for disease management. Through promoting health behaviors and mutual supports between the couple, CCMM seeks to enhance the couple’s disease management efficacy and improve their health as a whole [10]. CCMM roots in two theories, the Dyadic Model of Coping with Chronic Illness (DMCCI) and Social Cognitive Theory (SCT). DMCCI describes couples’ dyadic appraisal of the illness severity, ownership and management responsibility, and dyadic coping strategies [11]. Social Cognitive Theory (SCT) is an interpersonal level theory suggesting that we learn from the dynamic interaction between people (personal factors), their behavior, and their environments [12]. DMCCI elaborates on the couple’s coping process with chronic diseases, while SCT emphasizes the efficacy of disease management through collaboration. It is hypothesized that when the couple agrees on disease management as a joint responsibility, they may have more supportive behaviors and collective efficacy to facilitate their joint changes in behaviors and health conditions [13].

Despite developed theoretical basis of CCMM, its implementation remains challenging. By and large, barriers to implementing CCMM reported in previous studies can be grouped into two categories: low recruitment rates and poor adherence of couple-based interventions [14, 15]; as well as difficulties in forming couple collaboration [16, 17]. One qualitative study among Chinese couples indicates that the patients were reluctant to express feelings to their spouses, which might have hindered the formation of spousal empathy and common beliefs [18]. In addition, some spouses may refuse to be involved or get over-involved [16, 19]. Compared to young people, older couples may be more suitable for CCMM as they tend to spend more time together after retirement and have higher marital satisfaction with fewer conflicts in long-enduring marriages [11]. However, there is limited evidence on the barriers to implementing CCMM among older couples and their associations with different implementation outcomes.

To investigate the effect of CCMM on patients with type 2 diabetes mellitus, we conducted a multicentered randomized controlled trial (RCT) among 207 pairs of Chinese older couples in 14 community health centers in Guangzhou, China from August 2020 to May 2022 (Trial No. 2019-064) [20]. The coupled-based interventions consisted of four-week group education sessions and two-month telephone booster, delivered by community healthcare practitioners. Similar to previous couple-based trials, our study encountered difficulties especially low participation rate. We thus conducted the current study to explore the combinations of conditions necessary and sufficient (i.e., solutions) for CCMM’s implementation. Specifically, we utilized the qualitative comparative analysis (QCA) to disentangle key conditions of complex intervention [21] and generates plausible solutions (combination of conditions) [22], which would help improve implementing CCMM in diabetes management among older people.

## Methods

### Study design

This research consisted of a qualitative phase and a quantitative phase. In the qualitative phase, in-depth interviews were conducted among couples of the intervention group and corresponding practitioners, in the follow-up period of the multicentered RCT from January to April 2022 [20]. Key conditions identified through the interviews were analyzed then by QCA in the quantitative phase. The study was approved by the Ethics Committee of Sun Yat-sen University (2019-064).

### Study participants

Couples for the interviews were selected using a purposive sampling method. To cover heterogeneity and enhance representative of the participants, we first selected five communities based on the community’s average attendance rate (two above the mean and three below); among them, four couples per community were invited for the interview based on their joint attendance rate (two each above and below 50%), alongside the community’s healthcare practitioners. Couples were invited by telephone to conduct the interview together at the community health center. Detailed participants information has been described in previous articles [20]. The recordings and transcripts were kept strictly confidential and could only be accessed by the authors of this study.

### Qualitative phase: in-depth interviews

In order to better capture the influences of couple-based collaborative management, we designed an interview guide (see Appendix 1) based on the capability, opportunity, motivation–behavior (COM-B) model. COM-B model is an integrative theoretical model based on causal mechanisms to identify individual and environmental factors that influence behavioral change [23], and has been successfully applied to various health-related behaviors, such as smoking cessation [24] and vaccination [25, 26].

Each interview lasted 30 to 60 min and was conducted by three female researchers together (J.Z., CH.Y., YX.L.): a moderator who asked questions, a recorder, and an observer who captured the facial expressions, body movements, tone of the interviewee, and the interview settings. Audio recordings and interview notes were kept for all interviews. All the researchers were trained before the start of the study.

The interview data were coded verbatim and analyzed using NVivo 12.0 software. Two researchers (J.Z., CH.Y.) repeatedly read and coded the interview transcripts independently, prior to coding the interviews using thematic analysis. Each researcher described the meaning of the code they developed and developed the coding structure individually. Guided by the CCMM theoretical framework [20], codebooks were created and categorized into relevant themes and sub-themes, with newly identified themes added as separate components to the CCMM framework. We refined the codebook in an iterative loop. Two coders independently applied the codebook to the most recent interview data, compared codes, discussed differences with a third coder (YX.L.), and improved the codes. The final codebook was generated after the researchers had achieved at least 80% internal consistency in coding the same case. A prefinal thematic structure was reviewed at length and all researchers agreed on the expanded CCMM framework.

### Quantitative phase: QCA

Themes identified from the interviews were included as conditions in QCA. Each couple or healthcare practitioner was regarded as a case (the unit of QCA analysis) [27]. Fuzzy-set QCA (fs QCA) was used to explore possible combination(s) of conditions leading to CCMM implementation. Successful CCMM implementation (outcome) was defined as controlled glycemic level (HbA1c level ≤ 7.0%) from baseline to 6-month follow-up, in line with diabetes management clinical guideline [28]. The HbA1c level was collected from the multicentered RCT and tested by Daan Gene testing company. Using Boolean algebra [29], QCA is a set-theoretic approach to summarizing elements across cases, and establishing nonlinear causal relationships via identifying necessary conditions (i.e., always present if the outcome occurs), sufficient conditions (i.e. outcome always occurs if the condition exists), as well as combinations of conditions (i.e. solutions) [30, 31].

We examined each condition following the criteria developed by Ragin [27], and calibrated it to a truth table with values ranging from 0 to 1 to describe the relationship between solution and the outcome [32]. The condition necessity is quantified by a consistency level (indicating relationship strength, range 0 ~ 1) above 0.9, and solution sufficiency is quantified by a consistency level of 0.8 [27]. We also conducted robustness analyses by examining proportional reduction in inconsistency (measuring the probability of logical errors) and consistency levels. All analyses were conducted in fsqca software. Detailed assignment and analysis steps were provided in Appendix 2.

## Results

### Participants’ characteristics

Among the 20 couples and 5 healthcare practitioners initially invited, 12 couples (response rate: 60%) and 4 healthcare practitioners (response rate: 80%) agreed to be interviewed. No new themes emerged as the interviews progressed to the 12th couple and the 4th practitioner, which was considered as data saturation. As shown in Table 1, the interviewed couples had an average age of 68 years, with the patients on average one year older than their spouses. The average disease duration of patients was eight years. The majority of patients had below high school education and spouses had high school education. Couples were mostly retired and lived in the city. Most community healthcare practitioners were community physicians with an average of ten years of practicing experience.

**Table 1:** Characteristics of interviewees

Characteristics	Characteristics of type 2 diabetes patients, their spouse and community healthcare practitioners*		
	Patient	Spouse	Community healthcare practitioners
*n*	13	11	4
Female sex, *n*(%)	7(53.8)	5(45.5)	2(50)
Age(years), mean ± SD, range	67.7 ± 6.8, 59–79	66.8 ± 6.2, 57–76	N/A
Diabetes History(years), mean ± SD, range	8.3 ± 6.7, 2–22	N/A	N/A
Working with patients with diabetes(years), mean ± SD, range	N/A	N/A	9.8 ± 6.2, 4–18
Educational level, *n*(%)			
≤Primary school	4(30.8)	2(18.2)	N/A
Junior high school	6(46.2)	2(18.2)	N/A
Senior high school	3(23.1)	7(63.6)	N/A
Place of residence, *n* (%)			
City	9(69.2)	7(63.6)	N/A
Occupation, *n*(%)			
Pensioner/Flex job	10(76.9)	7(63.6)	
Security Guards		2(18.2)	
Industrial worker		1(9.1)	
Doctor			3(75)
Nurse	1(7.7)		1(25)

*A couple in the interview who both had diabetes

### Conditions of CCMM implementation identified through interviews

Based on the interviews, 6 themes and 26 sub-themes were identified. Detailed explanations, descriptions and quotations for each theme are provided in Table 2.

**Table 2:** Interview code and content

Themes		Sub-themes			Implementation barriers addressed in the theme	Supporting quotations
		Key word	Description	Information source (Number of mentions/ total number)		
**Implementation Process**						
*Evaluation and reflection of the trial implementation by community health care providers and the completion of each step.*		Community healthcare practitioners	Attitude toward trials and the ability to perform rigorously	Medical Staff Interviews (4/4), Couple Interviews (11/12), Attendance	1. Practitioners generally reported that older adult was difficult to educate and manage. 2. It is not feasible to change the deep-rooted habits and behaviors of the older patient in the short term. 3. Some of the Practitioners did not strictly control the quality, especially they did not emphasize the concept of “couple synergy”, which weakened the difference between the intervention group and the control group, and the specificity was not outstanding compared with other health education course.	*“Because the age of our participants in the course, the average would be 65 years old, I think. They are already elderly, and some of their perceptions of diseases are more fixed… The health education points that we say, they accept, may not change so easily, their glucose will not be controlled easily, and this is a longer process.” (Community health worker 2)* *" At that time it was the concept of couple synergy was not deliberately emphasized in their courses.” (Community health worker 3)*
		Implementation Steps		Medical Staff Interviews (4/4), Couple Interviews (11/12)		
		Difficulties faced by community healthcare practitioners	Difficulties that are still unsolvable through efforts	Medical Staff Interviews (4/4)		
		Trial differentiation	Whether to highlight the role and importance of “couples”	Medical Staff Interviews (2/4), Couple Interviews(3/12)		
**Couple’s roles**						
*The relationship basis of the patient with the spouse and other family members, the distribution of health management responsibilities between the couple.*		Role assignment	Who is the dominant party in life	Medical Staff Interviews (1/4), Couple Interviews (10/12)	1. Almost all patients and spouses agree on the independence centrality of health management and ignore the effect of spousal help. 2. Spousal disharmony significantly affects the willingness and ability of spouses to participate in patient health management.	*Wife: “Look at him, I have to manage him? He can do everything by himself”.* *Husband: “Manage by myself, measure glucose and diet are managed by myself”. (Couple 07, husband, patient; wife, spouse)* *“I feel very impatient, to serve a patient at such an old age… I’m barely able to take care of her, so I don’t have any energy to worry about you.” (Couple 06, husband, spouse; patient with hemiplegia)*
		Couple relationship		Medical Staff Interviews (1/4), Couple Interviews (7/12)		
		Patient independence centrality	The belief that a spouse’s help isn’t needed	Couple Interviews (11/12)		
		Spousal willingness to help		Couple Interviews (12/12)		
		Influence of other family members	Perceptions of how to treat patient health management	Couple Interviews (11/12)		
**Belief and perception**						
*Focus on patient and spouse perceptions of the disease and trial.*		Perceptions of the disease	Correctness and importance of knowledge about diabetes	Couple Interviews (11/12)	1. Diabetes is considered a “natural disease that comes with age” and is not given enough attention. 2. The vast majority of couples mention some wrong ideas and stick to them. 3. Adherence to the established exercise and diet practices, believing that the new methods are not as effective as the established self-management.	*“I don’t know much about it yet, anyway, I can eat or sleep, I don’t feel anything.” (Couple 08, wife, patient)* *“The doctor told me to take metformin three times a day, I just take it once, I don’t take so much, (I’m) worried about the liver (that there will be side effects).” (Couple 05, husband, patient)* *“Then I’d rather go to Baiyun Mountain exercise, that doctor, she told me many time but I didn’t come, I just think don’t bother me.” (Couple 02, wife, spouse)*
		Perceptions of the trial	Perceptions of health education and couple-based intervention	Couple Interviews (12/12)		
		Own opinion	Misconceptions that are difficult to change even after intervention	Medical Staff Interviews (2/4), Couple Interviews (5/12)		
		Long-term adherence	Long-held mindsets and lifestyle habits before intervention	Couple Interviews (8/12)		
**Objective obstacle**						
*Barriers to participation in the intervention or the occurrence of behavior change*	Economic burden			Medical Staff Interviews (1/4), Couple Interviews (6/12)	1. The physical discomfort of older adult such as being easily hungry and tired, disability, etc. can significantly hinder them from making changes in diet and exercise. 2. The content of the courses is too elaborate and strict, which makes it difficult to follow, and can cause fear and reluctance to implement or adhere to them.	*“She just has hemiplegia and can’t do much exercise right now, and it affects her glucose which is poorly controlled for a long time.” (Couple 07, husband, spouse)* *“Eating only a finger-sized amount of meat, it’ s funny. I didn’t refute it at the time, how could it be less like that.” (Couple 04, husband, patient)*
	Difficult to implement		Interventions that are too severe or too burdensome to implement	Couple Interviews (6/12)		
	Lack of culture		Unable to understand the content of the intervention	Couple Interviews (2/12)		
	Physiological hindrance			Couple Interviews (7/12)		
	Time and distance conflicts		There is an incompatibility of time or distance between affairs and intervention	Couple Interviews (6/12)		
**Subjective initiative**						
*Factors that promote or encourage behavior change*	Experience’		Spouses and others’ experiences	Couple Interviews (5/12)	1. Half of the patients treated their spouse’s reminders with rejection and exclusion. The vast majority of patients believe that diabetes management is their own problem and not their spouse’s, which also reduces their spouse’s desire to participate in health management.	*Wife: “(Diet) less oil and salt is healthier”.* *Husband: “Is it healthy? If it’s healthy, you’re still like this (physical condition)”? (Couple 04, both husband and wife are patients)*
	Spousal Persuasion			Couple Interviews (8/12)		
	Physiological feedback			Couple Interviews (10/12)		
	New knowledge		Affirming the correctness of knowledge of health education	Couple Interviews (10/12)		
	Trust in physicians			Couple Interviews (2/12)		
**Behavior change**						
*Behavioral changes occurring after the intervention*	Sole change			Couple Interviews (11/12)	1. Female patients are more likely to be the bearers of family life, and male spouses may give less support in life.	*Wife: “Every time I (help him) injection (insulin), he does not know, and he does not learn, director He said (patients) to learn how to inject, it should be their own task. I do not follow him 24 hours a day”.* *Husband: " So why you need a spouse? " (Couple 04, both husband and wife are patients)*
	Change with spousal assistance		Behavior change that requires spousal companionship, services, and substitution.	Couple Interviews (11/12)		
	Lack of behavior change			Couple Interviews (10/12)		

*Implementation process* showed the implementation dilemma from the practitioners’ perspective. The community healthcare practitioners all reported (4/4) that older adults had established inherent ways of thinking and behaving, and it was difficult to achieve rapid change in their long-term habits. In addition, they believed that outcomes largely relied on the patient’s adherence and the couple’s relationship than the intervention itself, meaning that the effectiveness of the intervention was limited by the couple’s willingness and capability to collaborate in diabetes management.

*Couple’s role in management* showed the distribution of management responsibility between the couple. Although the majority of spouses (11/12) were more or less involved in the patient’s diabetes management, patients still believed diabetes health management to be their responsibility. Female patients were more willing to accept their spouse’s company and advice, whereas male patients seemed to show more independence.

*Belief and perception* reflected the common misconceptions about diabetes mellitus in older adults. Most couples (10/12) attributed the causes of diabetes to age, occupational experience, and genetic factors. But their knowledge about normal glucose ranges or possible consequences associated with high or low blood glucose was inadequate, which may contribute to poor engagement and adherence of the couple.

*Subjective imitative*, and *objective obstacle* were two themes related to promoting and hindering factors of behavior changes required by the intervention. Care and reminders from spouses were the strongest motivations to generate positive behaviors, while illness and frailty were the most significant physical obstacles which impeded the capability of adhering to daily health management activities. In addition, patients also generally reported that caring for grandchildren took up too much of their time, and such conflicting commitments may result in the absence of intervention.

*Behavioral change* occurred after the couple-based intervention. Among 12 couples interviewed, 10 patients simultaneously generated sole behavioral change while receiving assistance from their spouses. And one patient only reported sole behavioral change with a complete absence of spousal assistance, and one patient’s diabetes management was completely dependent on spousal assistance. Daily glucose monitoring was the most difficult management behavior for older couples to conduct. Some couples found management behaviors required by the intervention were just too strict or complex for them to perform.

**Fig. 1:**
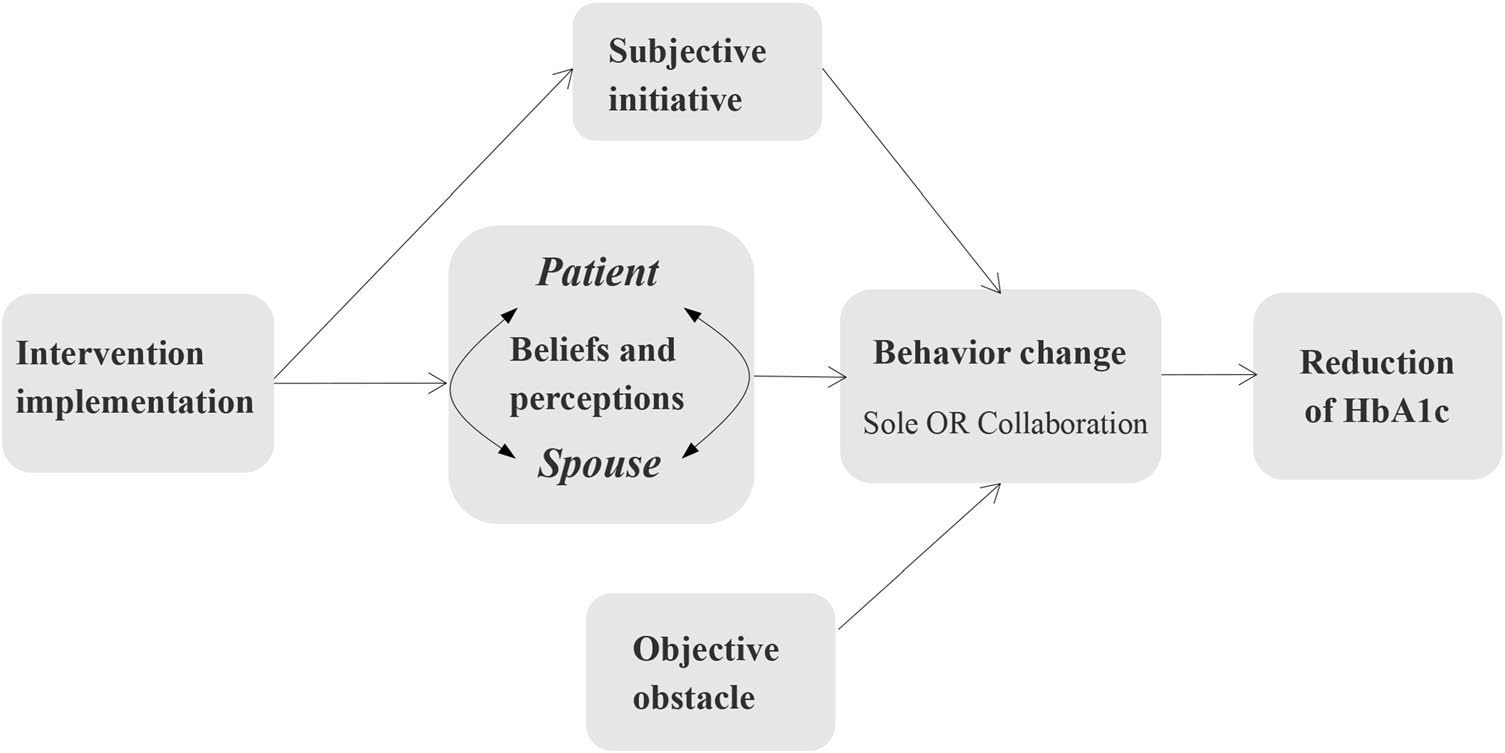
Expanded implementation framework of couple-based collaborative management model

Given the themes of implementation conditions identified above, the CCMM framework was expanded into Fig. 1. The expanded framework additionally highlighted the role of intervention implementation, subjective initiative, and objective obstacles on management behavior changes, which may then lead to HbA1c control.

### Solutions for successful CCMM implementation suggested by QCA

The hypothetical solution as in Fig. 1 was then validated via QCA and the necessity of the single conditions was displayed in Table 3. Judging by the consistency level of 0.90 criterion for a necessary condition [27], no single condition of Fig. 1 alone was necessary to control HbA1c. On the other hand, three combinations of conditions were identified as solutions for successful implementation. As shown in Table 4, cases fulfilling either solution had a 62.6% possibility of achieving a successful outcome, and the three solutions together explained 84.5% of the successful cases.

**Table 3:** Necessity of conditions within couple-based collaborative management model (CCMM) for HbA1c reduction

	Decreased HbA1c		Non-decreased HbA1c	
Condition	Consistency	Coverage	Consistency	Coverage
High-quality implementation process	0.511	0.540	0.488	0.460
~ High-quality implementation process	0.489	0.517	0.512	0.483
High couple collaboration	0.697	0.769	0.566	0.557
~High couple collaboration	0.598	0.606	0.765	0.693
Correct belief and perception	0.727	0.720	0.733	0.648
~Correct belief and perception	0.645	0.730	0.684	0.691
High subjective initiative	0.801	0.726	0.670	0.541
~High subjective initiative	0.494	0.626	0.661	0.748
High objective obstacle	0.677	0.670	0.728	0.644
~High objective obstacle	0.640	0.725	0.627	0.634

~ means the counterfactual analysis of the condition

**Table 4:** Solution (combination of conditions) in couple-based collaborative management model (CCMM) for HbA1c reduction

Condition	Solution		
	1	2	3
Implementation process	•	⊗	●
Couple’s roles	●	●	⊗
Belief and perception	●	●	⊗
Subjective initiative	•	⊗	●
Objective obstacle		⊗	●
Consistency	0.870	0.815	0.865
Raw coverage	0.254	0.263	0.252
Unique coverage	0.111	0.263	0.109
Solution consistency	0.626		
Solution coverage	0.845		

● indicates that the condition exists as a core condition

• indicates that the condition exists as a peripheral condition

⊗ indicates that the condition is absent, a blank indicates irrelevance

For *Solution 1* (a consistency level of 0.87, explained 25.4% of successful cases), the reduction of HbA1c was achieved via a combination of a high-quality intervention process, collaborative couple’s roles, correct belief, and perception as well as subjective initiative for CCMM. Similarly, *Solution 2* (a consistency level of 0.82, explained 26.3% of successful cases) also emphasized collaborative couple’s roles, correct belief, and perception, but with a low-quality intervention process and lacking initiative, this solution can only achieve the reduction of HbA1c in the absence of objective obstacles. Both solutions entailed the couple’s collaboration and correct belief and perception of diabetes and its management, and this collaborative effect helps the patients manage diabetes.

On the contrary, *Solution 3* (a consistency level of 0.86, explained 25.2% of successful cases) suggested a solution without the couple’s collaboration, such that high-quality intervention may nevertheless change the patient’s misunderstanding about diabetes, and promote self-motivation to overcome objective obstacles. For example, the spouse of couple 08 missed many couple-based intervention courses, which were attended by the patient alone. However, the courses provided instruments to patients for glucose monitoring, which motivated the patient to accomplish health management by herself.

## Discussion

The present study investigated conditions and solutions of implementing CCMM in Chinese older couples with type 2 diabetes mellitus. We found that the main conditions centered on implementation process, couple’s role in diabetes management, and their belief toward CCMM and ability to perform it. QCA further identified three solutions-either promoting couple collaborative management (Solution 1 & 2) or enhancing patient self-management (Solution 3).

### Implementation conditions of CCMM

Most of the identified conditions associated with couple’s role were in line with prior couple-based interventions, including marital discord [33], inappropriate forms of collaboration [17, 19], and discomfort with role change [18]. In addition, enacting a couple-based intervention requires coordinating participation of the couple pair, either one of them encountered objective obstacles such as distance and time [34], conflicting commitments [35], or financial difficulties [36] would hinder the implementation. Our study further contributes to the literature by identifying some unique implementation conditions of couple-based interventions among older Chinese couples, associated with older patients’ strong autonomy and low literacy. As regards strong autonomy, some older patients were just too stubborn to accept assistance from their spouses. It might result from older couples have already established a stable and deeply-rooted lifestyle, and formed an equilibrium in diabetes management over the long disease duration. Such that additional couple-based management may seem to be unnecessary, or difficult to achieve fundamental change through short-term interventions.

Moreover, the majority of our study participants were born between 1943 and 1965, generations with relatively poor education (i.e., lower than senior high school). The older couples’ low literacy may further result in their misconceptions about diabetes, and thus impede their understanding and application of couple-based interventions [37]. Our between-sex comparisons further showed that male patients were less likely to view diabetes care as a couple-shared responsibility, or to express concerns and emotions about diabetes to their spouses than their female counterparts. This may reflect that Chinese older men were used to characterize themselves as “tough guys” in life, thus would be shameful to show their distress or weakness, and discomfort with role change [18]. This gendered care pattern was also reported in American couples over food-related behavior changes [38]. Masculine gender roles were identified as a barrier, with husbands paying less attention to their wives’ health promotion activities [39]. It also may be a manner of protective buffering [17], such that protecting their spouses from negative emotions and avoiding arguments due to diabetes symptoms or management requirements.

Under the aforementioned circumstances, CCMM implementation would be even more difficult when the trust between older patients and their healthcare practitioners was low. Practitioners generally reported that it was hard to reason with older patients, let alone alter the older couple’s behaviors to obey the interventions. This frustration may in turn affect practitioners’ motivation to further implement CCMM. Previous studies have shown that Chinese residents’ trust in the healthcare system was low [40], especially in community healthcare centers [41]. In turn, patients’ mistrust may lead to practitioners’ negative attitudes [42], hence reducing the motivation and quality of the intervention implemented [43].

### Solutions for successful CCMM implementation

To overcome the implementation conditions identified above, we found three solutions leading to HbA1c control, which can be further grouped into couple collaborative management type and self-management type.

The couple collaborative management type is embodied by Solution 1 and 2, both emphasizing the core conditions of couple collaboration and the correct perception of diabetes management. Our interviews indicated that supportive behaviors from the spouses provided more care and assistance for the patients, and created a harmonious family atmosphere encouraging patients to be responsible for their health. Previous studies have also found that spouse engagement is strong social support leading to significant and lasting improvements in glycemic control [44]. Moreover, we noted that older couples’ correct perception of diabetes and its management was a prerequisite for them to form a beneficial collaboration. Similar findings have been reported previously that spouses with more knowledge about diabetes may associate with performing more supportive and collaborative behaviors [45]. In addition, our previous studies also indicated that couple pairs’ relative differences in sociodemographic characteristics should be taken into account in couple-based intervention, such as the spouse’s age in reference to the patient’s (i.e., younger, the same, or older than the patients) and retirement status [46].

Despite the similarity in couple collaborative management type, these two solutions differed by several conditions. A high-quality intervention process and subjective initiative to change were needed for Solution1; while a low-quality intervention process rendering no motivation to change, the absence of objective obstacles was essential for Solution 2. Taken together, Solution 2 reflects an ideal situation when older couples with adequate knowledge about diabetes and neglectable objective obstacle, formed collaborative relationships in daily diabetes management, regardless of the CCMM intervention. Solution 1, in contrast, indicates the importance of high-quality CCMM intervention to enhance older couples’ knowledge and motivation to counteract objective obstacles, which may be more commonly seen in real practice.

On the other hand, the self-management type suggests the control of HbA1c can also be achieved through motivating the patients alone. Although against our intention to promote the couple’s collaboration via the CCMM intervention, patients who joined the courses without the company of their spouses were still able to achieve satisfying management results. It is suspected two main reasons may lead to the unintended self-management type. First, CCMM interventions were misinterpreted by some practitioners. Our interviews with the practitioners indicated that few of them overlooked the emphasis should be on “dyadic coping” in diabetes management, while purely delivered the courses as regular “self-management” education. The fidelity of intervention implementation (i.e., the extent to which the intervention is delivered as intended) is critical to the successful translation of CCMM strategies into practice [47]. Second, the older couples’ education levels and marital quality also affected their willingness to collaborate. As discussed above, some couples’ poor health literacy may confine their ability to comprehend the intervention contents or adapt to the recommended way of co-management. Despite relatively good marital quality would be expected among older couples who participated in our study, uncooperative behaviors of the spouses were also common such as non-attendance of the courses. One couple even divorced during our study follow-up. Collaboration would be hard for couples with negative attitudes or undesirable collaboration modes such as strong independence centrality, which may even lead to negative affect on the physiological and psychological function of the couples [48, 49]. For these couple pairs, couple-based intervention may be not suitable [16].

### Implications for theory and practice

The present study extends the CCMM theoretical framework. We added new themes of implementation process, subjective initiative, and objective obstacles to CCMM, and highlighted the core conditions-the correct view of diabetes and collective efficacy, for the collaborative management type of couple. Older couples’ characteristics and marital quality should be taken into consideration. Furthermore, the original CCMM emphasizes collective behavior change. However, our study has extended this by proposing the other potential pathway for individual patient behavior change. As described in the DMCCI [11], it is possible that individual coping as well as dyadic coping may be involved simultaneously in different aspects of diabetes management. Future research on couple collaboration could further explore the relationship between self-management and co-management.

The practical contribution of this study is to provide evidence to apply tailored CCMM in accordance with couples’ preference and literacy. For couples who are willing to cope with diabetes together, enhancing couple collaboration and correct perception would be more likely to control diabetes successfully. For those who are not suitable for couple collaborative management, self-management plan should be preferred. In addition, intervention fidelity, adequate support, and incentives are important to help overcome setbacks during the intervention implementation. Apart from that, policymakers should strengthen the trust between physicians and patients, which would promote the improvement of physicians’ work attitudes and patients’ adherence.

### Strengths and limitations

Our study innovatively used QCA to systematically investigate the theoretical framework utilized in the intervention and identified core solutions of successful CCMM implementation. Nevertheless, several caveats require attention. First, our study sample was small and with limited representativeness. Participants with unsatisfying marital quality and poor adherence to our intervention may not respond to our study. Second, QCA is semiquantitative by nature, and the causal relationship was verified based on qualitative information. To minimize subjective judgment, we involved five well-trained researchers with a different research background in coding and assigning values and used the robustness analyses to test confidence in the proposed relationships. The further quantitative analysis would be insightful to complement the findings here. Last, some information was lost due to compressing the rich interview findings into several themes and subthemes. This loss of information is unavoidable to identify the core conditions, and the case elaboration was used to expand and recover this missing.

## Conclusions

Main barriers of CCMM implementation among Chinese older couples were patients’ strong autonomy, low literacy, and mistrust between practitioners and older patients. This multicomponent intervention should be flexible, as interventions are most effective when they are tailored to older couples’ characteristics and literacy (either collaborative management or self-management). For collaborative management, the emphasis should be put on correct belief and perception about diabetes and the couple’s collective efficacy; while for patients preferred self-management, high-quality interventions to improve knowledge and skills of diabetes care would be essential. Our findings contribute to the development of tailored program to achieve better chronic disease management in older couples with different barriers.

### Electronic supplementary material

Below is the link to the electronic supplementary material.

**Supplementary Material 1:** Interview outline

**Supplementary Material 2:** Qualitative comparative analysis (QCA) analysis steps

## Data Availability

The datasets used and/or analysed during the current study are available from the corresponding author on reasonable request.

## List of abbreviations

CCMM: Couple-based collaborative management model
DMCCI: Dyadic Model of Coping with Chronic Illness
HbA1c: glycated hemoglobin
QCA: qualitative comparative analysis
RCT: randomized controlled trial
SCT: Social Cognitive Theory
