# Supplementary material for: Conditions for successful implementation of couple-based collaborative management model of diabetes among community-dwelling older Chinese: a qualitative comparative analysis
Source: BMC Geriatr. 2023 Dec 11;23:832. doi: 10.1186/s12877-023-04565-y (PMC10712117; doi:10.1186/s12877-023-04565-y)
Supplement: Supplementary file 1 — Supplementary Material 1: Interview outline [file 12877_2023_4565_MOESM1_ESM.docx]

**Appendix 1: Interview outline**

**Interview opening statements**

Hi, I am Jing Zhang, a researcher from Sun Yat-sen University. First of all, thank you very much for being part of our interview process. You have been involved in our couple-based collaborative management project. The purpose of this interview is to get your thoughts and attitudes about the project and diabetes, and to better understand the practical problems you faced to help us improve it. This interview will be recorded, but the content will not be used for anything but our research. Before beginning, I would like to invite you to introduce yourself briefly, thank you!

Interview content

**1.For older couples** **with type 2 diabetes mellitus**

1. When was diabetes diagnosed, and could you recall the feel when you were diagnosed?
2. How did the family know the patient had been diagnosed with diabetes?
3. How does it affect your life?
4. To the best of your knowledge, what kind of disease do you think diabetes is?
5. How did you get involved in this project? Why did you decide to come?
6. What was your first impression of the project? And what was the impression after you actually participated in it?
7. How do couples manage diabetes together? How do children and other family members help patients manage diabetes?
8. What can you recall from the project implementation? What is the most impressive thing you mentioned?
9. What was the most difficult part of the process? In your opinion, how these difficulties could be solved?
10. Do you have any other suggestions?

**2.For community healthcare practitioners**

1. What are your responsibilities in this project?
2. How did you help patients with their diabetes health management before you started this project?
3. How do you view the role that spouses play in diabetes management? Has the perception of the role of spouses changed since implementation?
4. Can you describe exactly how you operated in the project? Did you strictly follow the management manual?
5. In practice, which aspect do you find the most difficult? How should this difficulty be solved?
6. How do you supervise patients and their spouses to follow the intervention content? How do you cope when they are unmotivated or unresponsive?
7. What do you think the significance of the couple co-management model compared to community physician-led or patient self-management? Is it meaningful?
8. Is it possible and necessary to promote it?
9. Do you have any other suggestions?
