# Supplementary material for: Conditions for successful implementation of couple-based collaborative management model of diabetes among community-dwelling older Chinese: a qualitative comparative analysis
Source: BMC Geriatr. 2023 Dec 11;23:832. doi: 10.1186/s12877-023-04565-y (PMC10712117; doi:10.1186/s12877-023-04565-y)
Supplement: Supplementary file 2 — Supplementary Material 2: Qualitative comparative analysis (QCA) analysis steps [file 12877_2023_4565_MOESM2_ESM.docx]

**Appendix 2**

Themes obtained from the qualitative study: implementation process, couple’s role, belief and perception, objective obstacle, and subjective initiative were used as conditions, and differences in patients’ glycemic levels (HbA1c) from baseline to 6-month follow-up was used as an outcome. Qualitative comparative analysis (QCA) was performed referring to Ragin's criteria ^[1]^.

**Step one: Calibration**

The conditions came from in-depth interviews and were calibrated using the assignment method by calculating the frequency of occurrence of each theme ^[2]^. The outcome was assigned and calibrated using the direct calibration method, the specific rules of which are as follows.

Implementation process: the mean value of the attendance rate of the intervention groups was used as a reference. If the attendance rate of the intervention group in the couple's community was lower than the mean value, a value of 0 was assigned, and if the attendance rate was higher than the mean value, a value of 1 was assigned.

Couple’s role: The ability of couples to cooperate was measured by four sub-themes: couple affection, patient autonomy, spouse willingness to participate, and family members' attitudes. Values were assigned according to a quintile based on the number of sub-themes present. A value of 1 was assigned to the presence of all four variables: good relationship, patient dependence, positive spousal willingness to participate, and family member support; 0 to the absence of all four variables. 0.25 to the presence of one of the variables, 0.5 to the presence of two of the variables, and 0.75 to the presence of three of the variables.

Belief and perception: The affective tendency and intensity of each sub-themes of beliefs and cognitions were evaluated according to a six-point scale with reference to the Generic Membership Evaluation Template (GMET) ^[3]^. Firstly, sub-themes are judged to be positive or negative, and then evaluated for the degree of emotion. The sub-themes were assigned a value of 0 for completely negative, 0.2 for strongly negative, 0.4 for mostly negative with some positive, 0.6 for mostly positive with some negative, 0.8 for strongly positive, and 1 for completely positive. The total assignment was obtained by integrating each sub-theme.

Objective obstacle: There are five categories of barriers: economic burden, difficult to implement, lack of culture, physiological hindrance, and time and distance conflict, which were assigned according to a six-point scale. A value of 1 was assigned to all five variables or to the presence of factors that seriously affect the patient's ability to care for himself/herself (e.g., hemiplegia, visual impairment), while none of the five variables was assigned a value of 0. One of the variables was assigned a value of 0.2, two of the variables were assigned a value of 0.4, three of the variables were assigned a value of 0.6, and four of the variables were assigned a value of 0.8.

subjective initiative: A total of five categories of motivation were included: experience, spousal persuasion, physiological feedback, new knowledge, and trust in physicians, which were assigned values according to a six-point scale. All five variables were assigned a value of 1, none of the five were assigned a value of 0. One of them was assigned a value of 0.2, two of them were assigned a value of 0.4, three of them were assigned a value of 0.6, and four of them were assigned a value of 0.8.

Outcome: HbA1c measurements minus baseline measurements at month six after enrollment in this study was used as the outcome variable. Since the successful outcome was defined as a decrease in HbA1c, the data were calibrated by the reverse calibration method. The HbA1c changes were ranked from smallest to largest, and values of 5% (fully affiliated), 95% (fully unaffiliated) and 50% (crossover point) were used as anchor points for direct calibration.

Table 1 presents the calibrated scores for all the conditions and outcome for each Interview participants, indicating the extent to which this condition is present for each solution, with 1 = condition fully present and 0 = condition fully not present.

TABLE 1: Calibrated data table for the qualitative comparative analysis

| Cases | implementation process | couple’s role | belief and perception | subjective initiative | objective obstacle | HbA1c changes |
| --- | --- | --- | --- | --- | --- | --- |
| 1 | 0 | 0.75 | 0.8 | 0.4 | 0 | 0.66 |
| 2 | 0 | 1 | 0.4 | 0.8 | 0.6 | 0.98 |
| 3 | 1 | 0.75 | 0.6 | 0.6 | 0.4 | 0.72 |
| 4 | 1 | 0.501 | 0.6 | 0.8 | 0.8 | 0.91 |
| 5 | 1 | 0.501 | 0.6 | 1 | 0.8 | 0.26 |
| 6 | 1 | 0 | 0.4 | 0.2 | 1 | 0.09 |
| 7 | 1 | 0.25 | 1 | 0.8 | 0 | 0.49 |
| 8 | 1 | 0 | 0.2 | 0.6 | 0.8 | 0.77 |
| 9 | 0 | 0.75 | 0.4 | 0.6 | 0.6 | 0.53 |
| 10 | 0 | 0.25 | 0.6 | 0.8 | 0.4 | 0.49 |
| 11 | 0 | 0.75 | 0.4 | 0.4 | 0.4 | 0.02 |
| 12 | 0 | 0.25 | 0.4 | 0 | 0.6 | 0.42 |

**Step 2: Comprehensive description of different cases**

The truth table is used to show the possible solutions of all variables. The conditions are k then 2^k^ configurations exist, in this study that is 2^5^, 32 configurations exist. A total of 12 cases were included in this study, which belonged to the small and medium-sized sample of QCA study, thus the minimum number of cases was set to 1 and the minimum consistency was set to 0.8. The PRI value is an index indicating the likelihood of homophily, and the larger the value, the smaller the likelihood of homophily. A threshold value of 0.5 was used to exclude the unstable cases. The final truth table was obtained as follows.

Table 2. Displays all solutions (combinations of conditions) based on different cases

| Cases | implementation process | couple’s role | belief and perception | subjective initiative | objective obstacle | HbA1c changes ^a^ | raw consist | PRI consist. ^b^ | SYM consist. ^c^ |
| --- | --- | --- | --- | --- | --- | --- | --- | --- | --- |
| 3 | 1 | 1 | 1 | 1 | 0 | 1 | 1.00 | 1.00 | 1.00 |
| 8 | 1 | 0 | 0 | 1 | 1 | 1 | 0.86 | 0.73 | 0.73 |
| 4,5 | 1 | 1 | 1 | 1 | 1 | 1 | 0.83 | 0.69 | 0.69 |
| 2,9 | 0 | 1 | 0 | 1 | 1 | 0 | 0.76 | 0.59 | 0.63 |
| 1 | 0 | 1 | 1 | 0 | 0 | 1 | 0.81 | 0.54 | 0.54 |
| 6 | 1 | 0 | 0 | 0 | 1 | 0 | 0.65 | 0.35 | 0.35 |
| 7 | 1 | 0 | 1 | 1 | 0 | 0 | 0.84 | 0.30 | 0.85 |
| 11 | 0 | 1 | 0 | 0 | 0 | 0 | 0.69 | 0.24 | 0.24 |
| 10 | 0 | 0 | 1 | 1 | 0 | 0 | 0.75 | 0.00 | 0.00 |
| 12 | 0 | 0 | 0 | 0 | 1 | 0 | 0.68 | 0.00 | 0.00 |

^a^ HbA1c: glycated hemoglobin.

^b^ PRI: Proportional reduction in inconsistency.

^c^ SYM: symmetrical version of PRI.

**Step 3: Boolean simplification**

A Boolean-based algorithm was used to perform a logical simplification of the truth table. The software fs qca 3.0 provides three kinds of solutions through sufficiency analysis: parsimonious solution (minimal formula generated with all logical residuals), intermediate solutions (minimal formula generated by adding only the logical remainder that matches the reality and the researcher's theoretical knowledge) and complex solutions (minimal formula generated without logical remainders), and the logical remainders mean that solutions without empirical examples. We chose to interpret the results through intermediate solutions (to identify solutions) and parsimonious solutions (to identify the core conditions). Before performing Boolean simplification, we performed a necessity analysis for each condition to prevent the necessity condition from being removed. With 0.90 as the consistency threshold, no individual condition was necessary to drive HbA1c reduction or non-reduction in older diabetic patients. The results of the sufficiency analysis with interpretation of the solutions are reported in the text.

Table 3. Result of necessity analysis

|  | Decreased HbA1c | | Non-decreased HbA1c | |
| --- | --- | --- | --- | --- |
| Condition | Consistency | Coverage | Consistency | Coverage |
| High-quality implementation process | 0.511 | 0.540 | 0.488 | 0.460 |
| ~ High-quality implementation process | 0.489 | 0.517 | 0.512 | 0.483 |
| High couple collaboration | 0.697 | 0.769 | 0.566 | 0.557 |
| ~High couple collaboration | 0.598 | 0.606 | 0.765 | 0.693 |
| Correct belief and perception | 0.727 | 0.720 | 0.733 | 0.648 |
| ~Correct belief and perception | 0.645 | 0.730 | 0.684 | 0.691 |
| High subjective initiative | 0.801 | 0.726 | 0.670 | 0.541 |
| ~High subjective initiative | 0.494 | 0.626 | 0.661 | 0.748 |
| High objective obstacle | 0.677 | 0.670 | 0.728 | 0.644 |
| ~High objective obstacle | 0.640 | 0.725 | 0.627 | 0.634 |

~ means the counterfactual analysis of the condition.

**Step 4: Robustness analysis**

Robustness analysis was used to illustrate the findings stability, we conducted a robustness analysis in two aspects. When the consistency threshold reduced from 0.8 to 0.75, a new solution has been added in addition to the above. When the PRI consistency increased from 0.5 to 0.6, sufficiency analysis still generated solutions1 and 3. Therefore, the results are robust.

[1] Ragin C C. Redesigning Social Inquiry:Fuzzy Sets and Beyond University of Chicago Press, 2008

[2] Speer J, Basurto X. Structuring the Calibration of Qualitative Data as Sets for Qualitative Comparative Analysis (QCA). Field Methods, 2010, 24

[3] Tóth Z, Henneberg S C, Naudé P. Addressing the ‘Qualitative’ in fuzzy set Qualitative Comparative Analysis: The Generic Membership Evaluation Template. Industrial Marketing Management, 2017, 63: 192-204
